# British Science during the War

**Published:** 1918-08-24

**Authors:** 


### BRITISH SCIENCE DURING THE WAR.

### Interesting Exhibits at King's College.

THE immediate object of the British Scientific Products Exhibition, opened at King's College on August 12, is to demonstrate the progress of British science during the war in its application to industry. A visit to the exhibition will convince anyone that this object has been very effectively achieved. But we may suppose that the organisers of the exhibition—the British Science Guild—have had a further aim before them in enforcing upon the State and also upon industrial capitalists the claim of scientific education and research to more liberal support than they have yet received from either. As Professor R. A. Gregory, chairman of the Organising Committee, remarked in addressing a gathering of journalists prior to the opening of the exhibition, the unhappy position of this country in this respect is indicated by the fact that we have only 5,000 students all told of higher science, whilst the United States have 40,000 and Germany 17,000. With the new spirit created by the war, it is perhaps not too much to hope that the holding of the exhibition will help to secure for science its rightful place in our educational and industrial system.

The exhibition is divided into fourteen sections, the titles of which will probably best explain its scope and character—Chemical Products and Processes, Physical Appliances, Electrical and Electro-magnetic Appliances, Optical Apparatus, Glass, Quartz, Refractories and Porcelain, Photographic Apparatus and Materials, Measuring and Mechanical Instruments, Surgical, Bacteriological, and Pathological Appliances, Papers, Textile Specialities, Illustrations and Typography, Natural Products, Food Production and Conservation, and Gas Traction.

### SUBSTITUTES FOR ENEMY PRODUCTS.

In the section Chemical Products and Processes, Boots Pure Drug Co., Ltd , the British Drug Houses Ltd., Messrs. Burroughs Wellcome and Co., Messrs. Evans Sons Lescher and Webb, Ltd., and Messrs. Thomas Kerfoot and Co. show specimens of numerous chemical products of clinical value which before the war were obtained exclusively, or almost exclusively, from enemy sources, such as iodine, aspirin, phenacetin, etc. The comprehensive character of these exhibits—a substitute would appear to have been produced for every German product of proved utility—testify to the enterprise of these British firms and the skill of their scientific advisers in rising to the occasion of the great emergency caused by the outbreak of the war.

### A GERMAN MONOPOLY BEATEN.

In the section Surgical, Bacteriological, and Pathological Appliances an equally gratifying object-lesson is given of the success with which British industry in alliance with science has proved itself the equal, if not the superior, of German inventiveness and adaptability. For instance, the manufacture of fluorescent and intensifying screens was up to 1914 practically a German monopoly. Messrs. Harry W. Cox and Co. show specimens of these screens, devised by their chemist, Dr. L. A. Levy, which are said by competent judges to be better than the finest that Germany has sent to this country. Then, again, artificial eyes used to be mostly imported from Germany, although they were often sold as being of British production. They were presumably bought on account of their price rather than quality, for they were badly made of poor material, and often cracked or fell to pieces. Mr. Albert E. Waters exhibits eyes of Birmingham make which, it is claimed, are most carefully made of the best material, so that the danger of breaking is eliminated and a much longer life is assured for them. Other interesting exhibits in this section are a cystoscope for the examination of the bladder (Allen and Hanburys, Limited), Captain Platt's instrument for bone surgery and other surgical instruments designed during the war (Mayer and Meltzer), improved stretcher (W. J. D. Roberts), and the papier-maché splint (Surgical Requisites Association). The Wellcome Physiological Research Laboratories have a noteworthy exhibit illustrating the various stages in the production of typical sera, vaccines, and tuberculins by means of specimens, diagrams, and photomicrographs. Charts show the reduction in mortality in the British Army on foreign service resulting from the employment of typhoid vaccines.

### WHEN PAPER IS PLENTIFUL.

There is a good show of optical apparatus and scientific glassware, including refractometers—of an improved type—that were formerly only "made in Germany," pocket lenses, dissecting instruments, bacteriological tubes, etc., all produced in this country since the beginning of the war as the result of research and experiment. In another section are to be seen food containers and food-preserving jars, milk and butter testers, and similar articles that have supplanted German goods. The Davis Gas Stove Co., Ltd., exhibit the Fruit and Vegetable Evaporator, a patent gas-heated cabinet stove for the drying of fresh fruit and garden produce for storage for after-season use—an innovation which has obtained the special commendation of the Food Production Department of the Ministry. A curious exhibit by Messrs. James Spicer and Sons, Ltd., demonstrates the many unsuspected uses to which paper can be put, including socks, waistcoats, bootlaces, towels, drinking-cups. Perhaps when the paper shortage is over and the need for economy remains the ideas thus exemplified will have considerable practical importance.

We have written enough to indicate the varied and interesting character of the exhibition for all who are concerned in the practical application of science to industry. The exhibition is announced to remain open until September 7, when King's College, it is to be presumed, will be again required for its normal purposes. But we should not be surprised if there is a public desire for an extension of this date, and we certainly think that, if possible, the exhibition should be transferred to one or two prominent centres.

